# Harnessing DLL3 inhibition: From old promises to new therapeutic horizons

**DOI:** 10.3389/fmed.2022.989405

**Published:** 2022-12-01

**Authors:** Diego Luigi Cortinovis, Francesca Colonese, Maria Ida Abbate, Luca Sala, Marco Meazza Prina, Nicoletta Cordani, Elisa Sala, Stefania Canova

**Affiliations:** ^1^Department of Medical Oncology, San Gerardo Hospital, Monza, Italy; ^2^School of Medicine and Surgery, University of Milano-Bicocca, Milano, Italy

**Keywords:** DLL3, small-cell lung cancer, rovalpituzumab tesirine, tarlatamab, molecular classification

## Abstract

Small-cell lung cancer (SCLC) is an aggressive neuroendocrine tumor with a high relapse rate, limited therapeutic options, and poor prognosis. The combination of chemotherapy and immune-checkpoint inhibitors brings a new therapeutic era, although the lack of predictive biomarkers of response reduces the efficacy of applying the treatment to the entire population of patients with SCLC. The lack of treatments able to bind to a specific target has always been a substantial difference to the non-small cell lung cancer (NSCLC) counterpart. Delta-like canonical Notch ligand 3 is a protein frequently overexpressed in SCLC and is therefore being explored as a potentially promising therapeutic target in high-grade neuroendocrine lung cancer. In this article, we critically review the activity and efficacy of old DLL3 inhibitors antibody-drug conjugate (ADC) and their failures through new compounds and their possible applications in clinical practice, with a focus on new molecular classification of SCLC.

## Introduction: The role of targeted therapies in SCLC

Small-cell lung cancer (SCLC) represents the most aggressive phenotype within the spectrum of all lung neuroendocrine tumors with rapid proliferation and chemoresistance to conventional antiblastic treatments. This results in poor prognosis in case of advanced stage at diagnosis (1, 2).

After more than 30 years, the first-line therapeutic paradigm for advanced stage has been changed by introducing new agents in combination with chemotherapy such as immune check-point inhibitors. This had led to an improvement of the median survival of these patients beyond 12 months (3, 4).

A major advance in modern oncological therapy in the field of lung neoplasms has been the identification of genetic factors, mostly linked to point mutations, deletions, insertions, translocations leading to the identification of tumor subtypes sensitive to molecularly targeted therapies. In addition to this, the evidence of some predictive markers of response to immunotherapy, although inaccurate, have determined the greatest impact on survival in NSCLC, changing the natural history of this disease (5, 6).

The lack of predictors of response to the most modern treatments leads to the failure of so-called precision medicine in SCLC.

For example, analysis of biomolecular factors of patients considered to be strongly benefiting from chemo-immunotherapy treatment (i.e., longer than 18 months) with atezolizumab did not lead to evidence of benefit in any subgroup, regardless of the status of the biomarkers analyzed (7).

The exploratory analysis conducted in the CASPIAN study points in the same direction. The association between the antigen presentation factors (HLA class 1/2 alleles) and the overall survival (OS) showed that the presence of the HLA-DQB1 ^*^ 03: 01 allele was associated with a longer OS in the durvalumab + tremelimumab and chemotherapy arm, but not in the other arms, providing a proof of concept for further studies in the future (8).

Although SCLC is characterized by numerous genomic alterations typically caused by a specific pathogenic noxa (cigarette smoke), the study of these alterations has not led to the determination of specific drugs. Within some seminal works, whole-exome sequencing of SCLC tumor surgical samples in a treatment-naïve population confirmed the already known genetic features of this disease, characterized by a high mutational burden (8.6 mut/Mb), universal loss-of-function mutations in TP53 and RB1 and rare actionable targetable mutations in KIT, PIK3CA, BRAF and amplification of FGFR1, SOX2 and MYC (9).

Recently, new avenues have opened up with the evaluation of different SCLC subtypes defined by the differential expression of four key transcription regulators: ASCL1, NeuroD1, YAP1 and POU2F3 (10). The reveal of potential therapeutic vulnerabilities of these subtypes may constitute a step forward in personalized SCLC medicine (11).

In oncology, an ideal target is generally a molecular alteration that is more highly expressed in tumor tissue than in healthy cells and represents a factor that substantially promotes and supports cell proliferation and that can be blocked by a specific therapy leading to cell apoptosis.

Delta-like canonical Notch ligand 3 (DLL3) is an inhibitory ligand of the Notch pathway that is highly conserved in developing lung neuroendocrine cells. Therefore, the resulting downregulation involves the growth of neuroendocrine tumor cells (12).

DLL3 is overexpressed on the cell surface of neuroendocrine tumor cells in about 80% of SCLCs, whereas it is normally expressed in the cytoplasmic area in healthy cells (13).

DLL3 expression is also regulated by the transcription factor achaete-scute homolog 1 (ASCL1) which, in recent works, has been recognized as an oncogenic driver whose alteration is present in about 60% of all SCLCs.

The differential expression profile of DLL3 in normal vs. oncogenic tissue makes this target particularly interesting from a therapeutic point of view (14).

In recent years, the establishment of DLL3 as a unique target in SCLC has accelerated the development of novel and promising therapeutic agents.

The history of drug development involving the manipulation of this target has led to mixed results starting with older antibody-drug conjugate (ADC) such as rovalpituzumab tesirine (ROVA-T) through renewed interest in immuno-oncological agents such as bispecific T-cell engager (BiTE) and chimeric antigen receptor T cell (CAR-T), with their attendant failures, to new compounds and their possible applications in clinical practice, with a focus on a new molecular classification of SCLC.

Further development of these drugs could lead to the beginning of a new era of specific and highly active therapies in the therapeutic strategy of SCLC and other neuroendocrine neoplasms (15).

In this review, we will critically focus on the development of treatments against DLL3 and their perspective in clinical practice.

## DLL3 expression and its role in high grade neuroendocrine lung cancer

DLL3 is a member of the Delta/Serrate/Lag2 (DSL) Notch receptor ligands, together with DLL1, DLL4, JAG1, and JAG2. Notch signaling is a highly conserved pathway involved in cell proliferation, differentiation, and apoptosis, which plays a pivotal role in the development of pulmonary neuroendocrine cells and is thus directly involved in the pathogenesis of certain tumors such as SCLC. DLL3 is an inhibitory ligand for the Notch receptor, normally located in the Golgi apparatus in healthy cells. DLL3/Notch binding prevents dislocation of the receptor on the cell surface and emerges on the cell membrane when it is pathologically overexpressed, resulting in aberrant growth of neuroendocrine tumor cells, including SCLC and large-cell neuroendocrine carcinoma (LCNEC) (13, 16). In a recent study, 63 patients with SCLC underwent immunohistochemistry (IHC) for DLL3: 52 patients (83%) were positive for DLL3 expression, with 20 patients (32%) showing high expression of DLL3 (positive in at least 50% of cancer cells) (17). DLL3 is not only involved in SCLC, but is also expressed in other tumor types of neuroendocrine origin, including melanoma, glioblastoma multiforme, small cell bladder cancer and castration-resistant prostate cancer (18). DLL3 expression is regulated by ASCL1, a transcription factor required for the proper development of pulmonary neuroendocrine cells, which is recognized as an oncogenic driver in ~60% of all SCLCs (14, 19). ASCL1 is one of four key transcription factors whose expression underlies the emerging molecular classification of SCLC. In contrast to the increasingly targeted drugs for patients with lung adenocarcinoma involving EGFR, ALK, ROS1, RET, BRAF, MET and NTRK, SCLC is still perceived and treated as a single disease, a “homogenous” entity without clinically relevant molecular subtypes. However, based on the expression of several neuroendocrine (NE) markers, such as chromogranin A (CHGA), synaptophysin (SYP), neural cell adhesion molecule 1 (NCAM1/CD56) and gastrin-releasing peptide (GRP) SCLC can be classified into neuroendocrine-high (NE-high) or neuroendocrine-low (NE-low) tumor. The NE-high and NE-low subtypes show distinct genetic alterations and a different susceptibility to immune checkpoint inhibitors (ICIs), suggesting that some sort of biological heterogeneity also exists for SCLC (20). The biological heterogeneity of SCLC has started to emerge through studies based mainly on preclinical models such as genetically engineered mouse models (GEMMs) and patient-derived xenografts (PDXs) (21). Recent genomic profiling studies have defined SCLC molecular subtypes based on the relative expression of key transcription regulators, including ASCL1, NeuroD1 (neurogenic differentiation factor 1), YAP1 (yes-associated protein 1), and POU2F3 (POU domain class 2 homeobox 3). Multiple independent researchers have proposed a consistent nomenclature for these SCLC subtypes: SCLC-A (A=ASCL1), SCLC-N (N=NeuroD1), SCLC-Y (Y=YAP1) and SCLC-P (P=POU2F3). SCLC-A and SCLC-N show high expression of NE markers. In contrast, SCLC-Y and SCLC-P are considered non-NE tumor subtypes. Whole genome sequencing (WGS) revealed an enormous mutational burden and a high number of genetic alterations that characterize each SCLC subtype more or less specifically: ~90% biallelic loss of TP53 and RB1, overexpression/amplification of cyclin D1 (CCD1), inactivation of cyclin dependent kinase inhibitor 2A (CDKN2A) and alteration in several genes involved in cell cycle regulation (CDK4/6), receptor kinase signaling (KIT, FGFR1), transcriptional regulation (CREBBP, MYC), apoptosis (SOX2, BCL2) and neuroendocrine differentiation/Notch signaling (22). Therefore, DLL-3 inhibitors now represent a potential therapeutic target approach in NE-high SCLC-A, underlining the importance of the emerging concept that heterogeneity in SCLC is primarily based on neuroendocrine differentiation, molecular subtype, and gene expression profile.

## The old journey: First generation DLL3 inhibitors

Rovalpituzumab tesirine is a first-in-class DLL3-targeted antibody-drug conjugate consisting of the humanized DLL3-specific IgG1 monoclonal antibody SC16, the DNA cross-linking agent SC-DR002 and a protease-cleavable linker that covalently binds SC-DR002 to SC16. Rudin et al. evaluated single-agent ROVA-T in SCLC or LCNEC with measurable progressive disease previously treated with one or two chemotherapeutic regimens, including a platinum-based regimen, in a first-in-human, open-label, phase I study (16). The primary objective of the study was to assess the safety of ROVA-T; secondary objectives were to characterize the pharmacokinetics and immunogenicity of ROVA-T, estimate its antitumor activity, and establish the recommended phase II dose and schedule. Eighty-two (82) patients were enrolled, 74 SCLC and 8 LCNEC (excluded from main endpoint analyses). All patients received at least one dose of ROVA-T. The maximum tolerated dose was reported to be 0.4 mg/kg every 3 weeks, but this dose was associated with an unacceptable level of delayed toxic effects. Consequently, the recommended dose and schedule of ROVA-T was defined as two cycles of 0.3 mg/kg every 6 weeks. Sixty-six (88%) patients reported treatment-related adverse events (ADs) of any grade, 28 (38%) patients of grade 3 or worse. Thrombocytopenia, serous effusions, and skin reactions were the most frequent treatment-related ADs of grade 3 or worse. Eighteen (22%) patients discontinued treatment due to ADs. Of the 65 patients assessable for activity analyses, 11 (17%) achieved a confirmed objective response and 35 (54%) stable disease. The median duration of response (DOR) was 5.6 months, and the median progression-free survival (mPFS) was 3.1 months. Median OS (mOS) was 4.6 months in the 68 patients treated with the active dose levels of ROVA-T. Considering the 29 DLL3-high patients, ten (35%) had a confirmed objective response, mPFS was 4.5 months, and mOS was 5.8 months. Udagawa and colleagues conducted a similar phase I, open-label, dose-escalation study among the Japanese population (23). SCLC patients, pre-treated with at least two systemic regimes, including one platinum-based regimen, received 2 doses of ROVA-T (0.2 or 0.3 mg/kg) every 6 weeks. Retreatment was allowed for patients who tolerated initial doses and achieved a disease control for at least 12 weeks after the last dose, but only one patient was retreated with ROVA-T. The primary objective was to assess safety and tolerability; secondary objectives were pharmacokinetics and preliminary efficacy. DLL3 expression was classified as high (≥75%), positive (≥25%), or negative (<25%) as IHC-based score. A total of twenty-nine (29) patients were enrolled, 6 in the 0.2 mg/kg cohort and 23 in the 0.3 mg/kg cohort. As expected, most patients (64%) expressed high-DLL3. All patients experienced at least one treatment-emergent adverse event (TEAE) of any grade; 15 patients reported AD of grade ≥3. These safety findings were accompanied by low activity: 3 (10%) patients achieved a confirmed partial response (PR). The median DOR was 3.0 months, mPFS was 2.2 months, and mOS was 5.8 months. All responders received 0.3 mg/kg ROVA-T and had tumors with high DLL3 expression. In patients with high DLL3 expression, mOS was 7.4 months compared to 5.1 months in non- high DLL3 patients (23). Despite the premise, ROVA-T was evaluated by Hann and colleagues in a phase I, multicenter, open-label study in a chemo-naïve population. After an initial chemotherapy cycle, ROVA-T was administered as monotherapy sequentially or in combination with cisplatin/carboplatin and etoposide (CE). The primary endpoint was safety, while secondary endpoints included efficacy and pharmacokinetic assessment of ROVA-T combined with CE. Based on preliminary safety and efficacy data, patients who received lower doses of ROVA-T (0.1 or 0.2 mg/kg) in combination with CE were selected for further evaluation. Drug-related TEAEs of any grade occurred in 14 (100%) patients, while serious adverse events (SAEs) were observed in 13 (93%) patients. Seven patients (50%) achieved a confirmed objective response rate (ORR), mPFS was 5.2 months and mOS was 10.3 months. These results do not suggest any efficacy benefit of frontline combination treatment with ROVA-T (24). Despite these discouraging results, the phase II TRINITY study was conducted in a population of relapsed or refractory SCLC. All patients received ROVA-T 0.3 mg/kg every 6 weeks for two cycles as initial treatment and a retreatment was allowed in patients who had benefited from the first cycle. The co-primary endpoints were ORR and OS. Secondary endpoints were DOR, disease control rate (DCR), and PFS. Three hundred thirty-nine patients were enrolled and received at least one dose of ROVA-T, most of them (70%) with DLL3-high. The ORR for the entire population was 12.4%, mPFS was 3.5 months, and mOS was 5.6 months with no significant difference in the DLL3-high subgroup. Almost all patients reported at least one TEAE, grade 3 or 4 TEAEs were observed in 179 (54%) patients. The most frequent severe ADs were in order of incidence: cutaneous reaction, edema, and pleural effusion (54 vs. 38 vs. 32%, respectively) (25). Lastly, the activity of ROVA-T was evaluated in two phase III studies. The TAHOE study was an open-label, two-to-one randomized, phase III study that compared the efficacy and safety of ROVA-T vs. topotecan in patients with DLL3-high SCLC progressed during or after first-line platinum-based chemotherapy. The primary endpoint was OS. The ROVA-T schedule used was the same as previously described in phase II studies. After enrolment of 444 patients, the study was stopped because OS with ROVA-T was shorter than with topotecan, and statistical tests for efficacy endpoints were not performed as originally planned. mOS was 6.3 months in the ROVA-T arm and 8.6 months in the topotecan arm; mPFS was 3.0 and 4.3 months, respectively. ORR was 15% in the ROVA-T arm vs. 21% in the topotecan arm, with grade 3 or higher ADs reported in the ROVA-T arm in 64% of patients (26). The second phase III study explored ROVA-T in the maintenance phase. MERU was a phase III randomized in a 1:1 ratio, double-blind, placebo-controlled study that enrolled patients with SCLC who had achieved disease control after four cycles of first-line platinum-based chemotherapy, measured as stable disease, partial response, or complete response according to RECIST v.1.1. The primary endpoints were PFS and OS in the population with DLL3-high tumors. After enrolment of 748 patients, the study was stopped early due to the OS-based futility analysis. In the high DLL3 population (61%), mOS was 8.5 months in the ROVA-T arm and 9.8 months in the placebo arm and ORR was 10 and 5%, respectively. The mOS for all randomized patients (secondary endpoint) was 8.8 months in the ROVA-T arm and 9.9 months in the placebo group. No significance was observed in the mOS of the population with DLL3-high tumors vs. DLL3-low. However, with regard to PFS, a favorable trend was observed for DLL3-high tumors. Overall, 343 (93%) patients in the ROVA-T arm and 304 (82%) in the placebo arm experienced at least one TEAE; TEAEs of grade greater than or equal to 3 occurred in 217 patients (59%) in the ROVA-T arm and 111 (30%) in the placebo arm (27). ROVA-T was also evaluated in combination with ICIs in a phase I–II study. The primary endpoint was to assess the safety and tolerability of administering ROVA-T in combination with nivolumab or nivolumab plus ipilimumab; the secondary endpoint was antitumor activity. Fourty two patients were enrolled, 30 in cohort 1 (nivolumab) and 12 in cohort 2 (nivolumab plus ipilimumab). Overall, 23 (55%) patients were DLL3 high. Four patients experienced dose-limiting toxicities (DLTs), of which one belonged to cohort 1 and three to cohort 2. All 42 patients reported one or more TEAEs, with 38 (91%) patients reporting grades ≥3. In cohort 1, the confirmed ORR was 27.6%, mPFS was 4.8 months, and mOS was 7.4 months; in cohort 2, the confirmed ORR was 36.4%, mPFS was 4.1 months, and mOS was 11.0 months. For the entire sample ORR was 30%, mOS 7.4 months, and PFS 4.2 months (28) (see Table 1). In all these trials, ROVA-T showed a unique toxicity profile, with pleural and pericardial effusion, peripheral oedema, cutaneous reaction, and thrombocytopenia among the most common ADs. The mechanism of these toxic effects is unclear, but the most likely explanation is premature linker lysis, which causes systemic release of the DNA cross-linking agent SC-DR002. Although ROVA-T was the first target therapy studied for SCLC, the lack of predictive biomarkers, the unique toxicity profile shown in all clinical studies, and the modest clinical activity led to the discontinuation of development of this drug (29).

**Table 1:** ROVA-T trials: Characteristics and results.

	**Phase**	**Treatment**	**Line**	**Patients (*n*)**	**DLL3 high (%)**	**ORR (%)**	**PFS (m)**	**OS (m)**	**any grade AE (%)**	**≥3 AE (%)**
Rudin (16)	I	ROVA-T	Pretreated	74	45	17	3.1	4.6	88	38
Udagawa (23)	I	ROVA-T	Pretreated	29	64	10	2.2	5.8	100	52
Hann (24)	I	CE plus ROVA-T (cohort 3)	First	14	100	50	5.2	10.3	100	93
Morgensztern (25)	II	ROVA-T	Pretreated	339	70	12.4	3.5	5.6	99	54
Blackhall (26)	III	ROVA-T vs. topotecan	Second	444	100	15	3	6.3	95	64
Johnson (27)	III	ROVA-T vs. placebo	Maintenance	748	61	10	/	8.5	93	59
Malhotra (28)	I-II	ROVA-T plus nivolumab/ROVA-T plus nivolumab and ipilimumab	Pretreated	42	55	30	7.4	4.2	100	91

CE, cisplatin/carboplatin and etoposide; DLL3, Delta-like canonical Notch ligand 3; ORR, objective response rate; PFS, progression-free survival; OS, overall survival; AE, adverse event; ROVA-T, rovalpituzumab tesirine.

## The new journey: Second generation DLL3 inhibitors

AMG 757 is a first-in-class bispecific T-cell engager antibody consisting of two domains. One domain binds the DLL3 on tumor cells and the other binds the CD3 part of the T-cell receptor. In this way, AMG757 connects DLL3-positive tumor cells and T-cells, producing both tumor cells lysis and T-cells activation. In addition, this binding causes the production of cytokines that overwhelm the immunosuppressive environment of the tumor (18, 30–32). The structure of the antibody allows an extended half-life of 9.8 days. *In vitro*, low doses of AMG 757 are sufficient to induce the killing of DLL-3 positive tumor cells by T-cells without effects on DLL3-negative cells, including normal cells. These pharmacokinetic properties allow delayed administrations in humans (30–32).

Giffin and colleagues evaluated AMG 757 efficacy in cell lines and xenograft mouse models derived from SCLC patients. They demonstrated that once-weekly administration of AMG 757 induces T-cell activation and expansion in xenograft and orthotopic mouse models derived from patients with SCLC tumors in. *In vitro*, AMG 757 leads to T-cell activation, the production of proinflammatory cytokine and the release of cytotoxic granules. Engaged T-cells kill SCLC cell lines, including those with low levels of DLL3 expression. *In vivo*, the authors evaluated the activity of AMG 757 in mouse models of patient-derived SCLC xenografts. Treatment with AMG 757 induced overall significant reduction in tumor volume. The activity was also evaluated in orthotopic SCLC models with weekly intravenous infusion. Similarly, AMG 757 treatment led to a significant reduction in tumor growth in these models. A single administration induced a significant increase in the number of human CD4+ and CD8+ T cells. In non-clinical toxicological studies, AMG 757 was well tolerated at the maximum dose of 4.5 mg/kg, confirming low DLL3 expression on normal cells (31).

Clinical experience in humans is also reassuring. An ongoing phase I study evaluated AMG 757 monotherapy in combination with anti-PD1 therapy and additional cytokine release syndrome (CRS) mitigation strategies in adult SCLC patients who had progressed or recurred after at least 1 platinum-based chemotherapy (NCT03319940). AMG 757 was administered intravenously once every 2 weeks at escalating doses up to 10 mg (0.003e10.0 mg). As of 7 august 2020, the study enrolled 40 patients with a median age 64 (44–80). Preliminary results from the monotherapy arm showed a median treatment duration of 6.1 weeks (0.1–59.4). AEs were reported in 39 (97.5%) patients and 4 (10%) discontinued treatment due to such effects. 32 (80%) were treatment-related, including 7 (17.5%) grade ≥3 and 1 (2.5%) grade 5 pneumonitis. Cytokine release syndrome (CRS) occurred in 18 (45%) patients, grade 1 or 2, none grade 3. The symptoms of CRS were fever and hypotension, occurred within 24 h of the first two doses (or during the first 24 h) and were reversible. There were no reports of interruption or discontinuation of treatment due to CRS. A confirmed partial response (PR) was observed in 6 (15.8%) patients and stable disease in 11 (28.9%) patients. Patients with confirmed PR were mostly heavily pre-treated with a median of 2 (1–4) prior lines of therapy. They had a DOR between 1.9 and 9.4 months. Tumor shrinkage occurred irrespective of DLL3 expression (range 55–300) (33). The trial is still active and recruiting.

Another novel therapy targeting DLL3 is AMG 119, a chimeric antigen receptor T cell (CAR-T). T cells are taken from the patient and genetically modified *ex vivo* to express a chimeric antigen receptor that targets DLL3. Subsequently, cytotoxic T-cells are re-administered to the patient to recognize and kill DLL3-positive cells. Unlike AMG 757, AMG 119 can induce long-lasting antitumor activity with a single administration (34).

Preclinical data have shown that AMG 119 has high potency and specificity for DLL3-positive tumor cells. *In vitro*, AMG 119 is shown to enhance T-cell cytotoxic activity and pro-inflammatory cytokine production. *In vivo*, AMG 119 induces tumor shrinkage in xenograft models (34).

Clinical data are immature. NCT03392064 is an open-label, phase I study evaluating the safety and tolerability of AMG 119. Secondary endpoints include ORR, PFS and OS. Eligible patients are adult patients with SCLC that has progressed after at least one platinum-based chemotherapy. AMG 119 is administered intravenous once. The trial is currently suspended.

## Discussion and future perspectives

Precise and effective therapy for SCLC represents an unmet medical need. Some progress has been made using modern technologies and next generation sequencing (NGS), but a thorough understand of the biology of SCLC is crucial.

DLL3 is an atypical ligand of the Notch receptor family that is found on the surface of tumor cells and in over 80% of SCLC. It should be noted that expression in normal lung tissue is low or null. The Notch pathway is associated with cancer proliferation and DLL3 participates in neuroendocrine tumorigenesis. Moreover, DLL3 is associated with a poor prognosis, particularly in some rare neuroendocrine subtypes (35).

Based on the high DLL3 expression in SCLC and LCNEC, DLL3 represents an interesting and novel targeted therapy.

In recent clinical trials, ROVA-T, a DLL3-targeting Ab-drug conjugate, has been tested as a novel antitumor drug. However, the phase III trials TAHOE and MERU (26, 27) demonstrated a shorter OS than standard therapy. Consequently, its development was permanently discontinued in August 2019. The absence of predictive biomarkers was a reason for the failure of Rova-T development. DLL3 expression was evaluated as biomarker, but while an enrichment of responses was observed in early studies in DLL3-high tumors, these results were not confirmed in phase 3 trials, although TAHOE trial enrolled only DLL3 high patients, thus the predictive role of DLL3 as biomarker was not tested in the same way as earlier trials (26). The combination of Rova-T with nivolumab + ipilimumab or nivolumab in the case of progressive disease (NCT03026166) was also discontinued after the DLT evaluation phase of the cohort (28).

Translational research is also investigating possible mechanisms of resistance to Rova-T, but there are currently no clinical implications (36).

Antibody drug conjugates are among the fastest growing drug classes in oncology; for example, the recent evolution in ADCs is evident in breast cancer; DESTINY-breast03 trial compared the efficacy and safety of trastuzumab deruxtecan (T-DXd), an ADC that combines the humanized anti-HER2 mAb trastuzumab with the topoisomerase inhibitor deruxtecan *via* a protease-cleavable peptide linker, with those of trastuzumab emtansine (T-DM1), an ADC composed of the anti-HER2 mAb trastuzumab connected to the microtubule inhibitor emtansine *via* a noncleavable linker, in patients with advanced HER2 positive breast cancer previously treated with trastuzumab and a taxane. In these patients the risk of disease progression or death was lower among who received trastuzumab deruxtecan than among who received trastuzumab emtansine (37). In this way, future development of rovalpituzumab, as mAb targeting DLL3 in the structure of an ADC, could include a different and more consistent linker, an increasing payload loading, novel and more powerful payloads or more innovative payloads that could overcome resistance to previous therapies (38).

Despite the discontinuation of ROVA-T development, new molecules targeting DLL3, such as near-infrared photoimmunotherapy, AMG 757, and AMG 119, have been explored with some promising data.

AMG 757 is a bispecific T-cell engager (BiTE). As bispecific recombinant proteins that target a T-cell surface molecule and a tumor-specific surface antigen, they promote T-cell adherence and anti-tumor response through an MHC-independent strategy (39). AMG 757 alone and in combination with pembrolizumab is being evaluated in a phase I study (NCT03319940) and is also being evaluated in combination with AMG 404 in a phase I/II study (NCT04885998). In addition, a phase II trial (NCT05060016) is ongoing in subjects with pre-treated, relapsed/refractory SCLC, in which tarlatamab, a half-life extended bispecific T-cell engager (HLE BiTE immune therapy) targeting DLL3, is being evaluated.

HPN328 is a tri-specific, half-life extended, T-cell engager targeting DLL3 and designed to minimize off-target toxicities. Interim results from an ongoing phase 1/2a study (NCT04471727) in patients with small cell lung cancer and other neuroendocrine cancers have shown promising results with regard to toxicity, and dose escalation is ongoing (40).

AMG 119 is a therapy based on CAR-T cells targeting DLL3. A phase I study (NCT03392064) was conducted in relapsed/refractory SCLC (currently suspended).

Specifically, Chen et al. (41) investigated the efficacy of DLL3-targeted bispecific antibody and CAR-T cells alone or in combination with immunotherapy.

The bispecific antibody and CAR-T showed activity in blocking the tumor growth *in vivo*. The association with the PD-1 inhibitor increased the activity of the DLL3 bispecific antibody, but not that of CAR-T cells. Although the results are rather encouraging, further studies are needed to verify this possible approach.

A new type of therapy, the near-infrared photoimmunotherapy has been providing intriguing results. Near-infrared photoimmunotherapy is an anticancer treatment technology that uses an Ab-photosensitizer conjugate followed by exposure to near-infrared light to damage cancer cells (42).

Incubating cells with ROVA-IR700 (ROVA-T conjugated with an IR700 photosensitizer) resulted in significant cell lysis upon exposure to near-infrared light. ROVA-IR700 has also been shown to shrink xenografts in mice (42).

Recently, another interesting therapeutic approach is radioimmunotherapy for SCLC. It consists of radiolabeling the anti-DLL3 antibody SC16 with the therapeutic radioisotope Lu-177 that emits beta particle. [^177^Lu] Lu-DTPA-CHX-A″-SC16 binds to DLL3 on SCLC cells and delivers targeted radiotherapy into the cancer cells, preserving healthy tissue.

A systemic radioimmunotherapy strategy employing a monoclonal antibody with high specificity for DLL3 is the basis of a proof-of-principle study conducted by Tully and colleagues in tumor-bearing mice (43).

The study investigated the preclinical efficacy and toxicity of 177Lu-labeled SC16 for the treatment of human SCLC in tumor-bearing mice. The results show impressive efficacy in mouse models of subcutaneous xenograft of SCLC, with moderate and transient hematologic toxicity and no significant hepatotoxicity.

These findings support [^177^Lu] Lu-DTPA-CHX-A″-SC16 as a potential development for clinical translation. Moreover, the possibility of using 89Zr-immunoPET to identify who would benefit more from targeted radioimmunotherapy with [^177^Lu]Lu-DTPA-CHX-A″-SC16 could represent a clinically meaningful opportunity (44).

Although the results are preliminary and need to be confirmed in further studies, they are appealing.

More recently, immunotoxin therapy is becoming a promising way to treat cancer. Ataee el al. (45) have designed two recombinant immunotoxins against DLL3 containing single-chain variable fragment rovalpituzumab antibody, which will require further experimental analysis.

All the above-mentioned findings are relevant to provide progress in the treatment of SCLC. Preclinical and clinical data show some encouraging outcomes.

The development of novel targeted therapies in SCLC is crucial and extremely challenging. The role of these drugs, alone or in combination with immunotherapy, radiotherapy, or other molecules, is being studied and it is hoped that they will change the scenario of SCLC treatment.

As SCLC is still a deadly disease, more attention should be paid to improving its therapeutic strategy. Strategies include advances in genomic profiling and biological pathways to identify potential tailored therapies and novel molecular targeted therapies. With regard to surface molecules, the identification of an affective antibody drug conjugate could be an attractive therapeutic target in the future, as well as radioimmunotherapy.

The outcomes of ongoing clinical trials and future research could contribute to breakthroughs in the treatment of SCLC.

## Author contributions

DC and SC designed the work. FC, MA, LS, MM, NC, ES, and SC contributed to data analysis and interpretation. MM drafted the first draft. All authors critically revised the work for important intellectual content. All authors approved the final version and agree to be accountable for all aspects of the work in ensuring that questions related to the accuracy or integrity of any part of the work are appropriately investigated and resolved.

## Conflict of interest

The authors declare that the research was conducted in the absence of any commercial or financial relationships that could be construed as a potential conflict of interest.

## Publisher's note

All claims expressed in this article are solely those of the authors and do not necessarily represent those of their affiliated organizations, or those of the publisher, the editors and the reviewers. Any product that may be evaluated in this article, or claim that may be made by its manufacturer, is not guaranteed or endorsed by the publisher.

## Abbreviations

ADC: Antibody-drug conjugate
AE: Adverse event
ASCL1: achaete-scute homolog 1
BiTE: Bispecific T-cell engager
CAR-T: Chimeric antigen receptor T cell
CE: Cisplatin/carboplatin and etoposide
CRS: Cytokine release syndrome
DCR: Disease control rate
DLL3: Delta-like canonical Notch ligand 3
DLT: Dose limiting toxicities
DOR: Duration of response
DSL: Delta/Serrate/Lag2
ICI: Immune checkpoint inhibitor
IHC: Immunohistochemistry
LCNEC: Large-cell neuroendocrine carcinoma
mOS: Median overall survival
mPFS: Median progression-free survival
NE: neuroendocrine
NeuroD1: Neurogenic differentiation factor 1
NSCLC: Non-small-cell lung cancer
NGS: Next generation sequencing
ORR: Objective response rate
OS: Overall survival
PFS: Progression-free survival
POU2F3: POU domain class 2 homeobox 3
PR: Partial response
ROVA-T: Rovalpituzumab tesirine
SAE: Serious adverse event
SCLC: Small-cell lung cancer
T-DM1: trastuzumab emtansine
T-DXd: trastuzumab deruxtecan
TEAE: Treatment-emergent adverse event
YAP1: Yes-associated protein 1.

## References

[1] Denninghoff V Russo A de Miguel-Pérez D Malapelle U Benyounes A Gittens A. Small cell lung cancer: state of the art of the molecular and genetic landscape and novel perspective. Cancers (Basel). (2021) 13:1723. 10.3390/cancers13071723 33917282 PMC8038650

[2] Gazdar AF Bunn PA Minna JD. Small-cell lung cancer: what we know, what we need to know and the path forward. Nat Rev Cancer 2017 1712. (2017) 17:725–37. 10.1038/nrc.2017.87 29077690

[3] Horn L Mansfield AS Szczesna A Havel L Krzakowski M Hochmair MJ. First-line atezolizumab plus chemotherapy in extensive-stage small-cell lung cancer. N Engl J Med. (2018) 379:2220–9. 10.1056/NEJMoa1809064 30280641

[4] Goldman JW Dvorkin M Chen Y Reinmuth N Hotta K Trukhin D. Durvalumab, with or without tremelimumab, plus platinum-etoposide versus platinum-etoposide alone in first-line treatment of extensive-stage small-cell lung cancer (CASPIAN): updated results from a randomised, controlled, open-label, phase 3 trial. Lancet Oncol. (2021) 22:51–65. 10.1016/S1470-2045(20)30539-8 33285097

[5] Wang M Herbst RS Boshoff C. Toward personalized treatment approaches for non-small-cell lung cancer. Nat Med. (2021) 27:1345–56. 10.1038/s41591-021-01450-2 34385702

[6] Grant MJ Herbst RS Goldberg SB. Selecting the optimal immunotherapy regimen in driver-negative metastatic NSCLC. Nat Rev Clin Oncol. (2021) 18:625–44. 10.1038/s41571-021-00520-1 34168333

[7] Liu SV Reck M Mansfield AS Mok T Scherpereel A Reinmuth N. Updated overall survival and pd-l1 subgroup analysis of patients with extensive-stage small-cell lung cancer treated with atezolizumab, carboplatin, and etoposide (IMpower133). J Clin Oncol. (2021) 39:619–30. 10.1200/JCO.20.01055 33439693 PMC8078320

[8] Garassino MC Shrestha Y Xie M Lai Z Spencer S Dalvi T. MA16. 06 Durvalumab ± Tremelimumab + Platinum-Etoposide in 1L ES-SCLC: Exploratory Analysis of HLA Genotype and Survival in CASPIAN. J Thorac Oncol. (2021) 16:S939. 10.1016/j.jtho.2021.08.198

[9] George J Lim JS Jang SJ Cun Y Ozretic L Kong G. Comprehensive genomic profiles of small cell lung cancer. Nature. (2015) 524:47–53. 10.1038/nature14664 26168399 PMC4861069

[10] Rudin CM Poirier JT Byers LA Dive C Dowlati A George J. Molecular subtypes of small cell lung cancer: a synthesis of human and mouse model data. Nat Rev Cancer. (2019) 19:289–97. 10.1038/s41568-019-0133-9 30926931 PMC6538259

[11] Poirier JT George J Owonikoko TK Berns A Brambilla E Byers LA. New Approaches to SCLC Therapy: From the Laboratory to the Clinic. J Thorac Oncol. (2020) 15:520–40. 10.1016/j.jtho.2020.01.016 32018053 PMC7263769

[12] Morimoto M Nishinakamura R Saga Y Kopan R. Different assemblies of Notch receptors coordinate the distribution of the major bronchial Clara, ciliated and neuroendocrine cells. Development. (2012) 139:4365–73. 10.1242/dev.083840 23132245 PMC3509731

[13] Saunders LR Bankovich AJ Anderson WC Aujay MA Bheddah S Black K. A DLL3-targeted antibody-drug conjugate eradicates high-grade pulmonary neuroendocrine tumor-initiating cells *in vivo*. Sci Transl Med. (2015) 7:302ra136. 10.1126/scitranslmed.aac9459 26311731 PMC4934375

[14] Borromeo MD Savage TK Kollipara RK He M Augustyn A Osborne JK. ASCL1 and NEUROD1 reveal heterogeneity in pulmonary neuroendocrine tumors and regulate distinct genetic programs. Cell Rep. (2016) 16:1259. 10.1016/j.celrep.2016.06.081 27452466 PMC4972690

[15] Yao J Bergsland E Aggarwal R Aparicio A Beltran H Crabtree JS. DLL3 as an Emerging Target for the Treatment of Neuroendocrine Neoplasms. Oncol. August. (2022) 27:940–51. 10.1093/oncolo/oyac161 35983951 PMC9632312

[16] Rudin CM Pietanza MC Bauer TM Ready N Morgensztern D Glisson BS. Rovalpituzumab tesirine, a DLL3-targeted antibody-drug conjugate, in recurrent small-cell lung cancer: a first-in-human, first-in-class, open-label, phase 1 study. Lancet Oncol. (2017) 18:42–51. 10.1016/S1470-2045(16)30565-4 27932068 PMC5481162

[17] Tanaka K Isse K Fujihira T Takenoyama M Saunders L Bheddah S. Prevalence of Delta-like protein 3 expression in patients with small cell lung cancer. Lung Cancer. (2018) 115:116–20. 10.1016/j.lungcan.2017.11.018 29290251

[18] Owen DH Giffin MJ Bailis JM Smit MAD Carbone DP He K. DLL3: an emerging target in small cell lung cancer. J Hematol Oncol. (2019) 12:61. 10.1186/s13045-019-0745-2 31215500 PMC6582566

[19] Augustyn A Borromeo M Wang T Fujimoto J Shao C Dospoy PD. ASCL1 is a lineage oncogene providing therapeutic targets for high-grade neuroendocrine lung cancers. Proc Natl Acad Sci U S A. (2014) 111:14788–93. 10.1073/pnas.1410419111 25267614 PMC4205603

[20] Schwendenwein A Megyesfalvi Z Barany N Valko Z Bugyik E Lang C. Molecular profiles of small cell lung cancer subtypes: Therapeutic implications. Mol Ther - Oncolytics. (2021) 20:470–83. 10.1016/j.omto.2021.02.004 33718595 PMC7917449

[21] Baine MK Hsieh MS Lai WV Egger JV Jungbluth AA Daneshbod Y. Small Cell Lung Carcinoma Subtypes Defined by ASCL1, NEUROD1, POU2F3 and YAP1: Comprehensive Immunohistochemical and Histopathologic Characterization. J Thorac Oncol. (2020) 15:1823. 10.1016/j.jtho.2020.09.009 33011388 PMC8362797

[22] Lum C Alamgeer M. Technological and therapeutic advances in advanced small cell lung cancer. Cancers. (2019) 11:1570. 10.3390/cancers11101570 31619019 PMC6826371

[23] Udagawa H Akamatsu H Tanaka K Takeda M Kanda S Kirita K. Phase I safety and pharmacokinetics study of rovalpituzumab tesirine in Japanese patients with advanced, recurrent small cell lung cancer. Lung Cancer. (2019) 135:145–50. 10.1016/j.lungcan.2019.07.025 31446987

[24] Hann CL Burns TF Dowlati A Morgensztern D Ward PJ Koch MM. A phase 1 study evaluating rovalpituzumab tesirine in frontline treatment of patients with extensive-stage SCLC. J Thorac Oncol. (2021) 16:1582–8. 10.1016/j.jtho.2021.06.022 34242790

[25] Morgensztern D Besse B Greillier L Santana-Davila R Ready N Hann CL. Efficacy and safety of rovalpituzumab tesirine in third-line and beyond patients with DLL3-expressing, relapsed/refractory small-cell lung cancer: results from the phase II TRINITY study. Clin Cancer Res. (2019) 25:6958–66. 10.1158/1078-0432.CCR-19-1133 31506387 PMC7105795

[26] Blackhall F Jao K Greillier L Cho BC Penkov K Reguart N. Efficacy and safety of rovalpituzumab tesirine compared with topotecan as second-line therapy in DLL3-high SCLC: results from the phase 3 TAHOE study. J Thorac Oncol. (2021) 16:1547–58. 10.1016/j.jtho.2021.02.009 33607312

[27] Johnson ML Zvirbule Z Laktionov K Helland A Cho BC Gutierrez V. Rovalpituzumab tesirine as a maintenance therapy after first-line platinum-based chemotherapy in patients with extensive-stage-SCLC: results from the phase 3 MERU study. J Thorac Oncol. (2021) 16:1570–81. 10.1016/j.jtho.2021.03.012 33823285

[28] Malhotra J Nikolinakos P Leal T Lehman J Morgensztern D Patel JD. A phase 1-2 study of rovalpituzumab tesirine in combination with nivolumab plus or minus ipilimumab in patients with previously treated extensive-stage SCLC. J Thorac Oncol. (2021) 16:1559–69. 10.1016/j.jtho.2021.02.022 33652156

[29] AbbVie Discontinues Rovalpituzumab Tesirine (Rova-T) Research and Development Program | AbbVie News Center. Available online at: https://news.abbvie.com/news/press-releases/abbvie-discontinues-rovalpituzumab-tesirine-rova-t-research-and-development-program.htm (accessed September 8, 2022).

[30] Giffin M Cooke K Lobenhofer E Friedrich M Raum T Coxon A. P3. 12-03 Targeting DLL3 with AMG 757, a BiTE® Antibody Construct, and AMG 119, a CAR-T, for the Treatment of SCLC. J Thorac Oncol. (2018) 13:S971. 10.1016/j.jtho.2018.08.1826

[31] Giffin MJ Cooke K Lobenhofer EK Estrada J Zhan J Deegen P. AMG 757, a half-life extended, DLL3-targeted bispecific T-cell engager, shows high potency and sensitivity in preclinical models of small-cell lung cancer. Clin Cancer Res. (2021) 27:1526–37. 10.1158/1078-0432.CCR-20-2845 33203642

[32] Giffin MJ Lobenhofer EK Cooke K Raum T Stevens J Beltran PJ. Abstract 3632: BiTE® antibody constructs for the treatment of SCLC. (2017) 2017:3632. 10.1158/1538-7445.AM2017-3632

[33] Owonikoko T Boyer M Johnson M Govindan R Rodrigues L Blackhall F. OA11. 03 A Phase 1 Study of AMG 757, Half-Life Extended Bispecific T-Cell Engager (BiTE®)Immune Therapy Against DLL3, in SCLC. J Thorac Oncol. (2021) 16:S126. 10.1016/j.jtho.2021.01.313

[34] Byers LA Chiappori A Smit M-AD. Phase 1 study of AMG 119, a chimeric antigen receptor (CAR) T cell therapy targeting DLL3, in patients with relapsed/refractory small cell lung cancer (SCLC). (2019) 37:TPS8576. 10.1200/JCO.2019.37.15_suppl.TPS8576

[35] Thomas A Takahashi N Rajapakse VN Zhang X Sun Y Ceribelli M. Therapeutic targeting of ATR yields durable regressions in small cell lung cancers with high replication stress. Cancer Cell. (2021) 39:566–579.e7. 10.1016/j.ccell.2021.02.014 33848478 PMC8048383

[36] Rath B Plangger A Krenbek D Hochmair M Stickler S Tretter V. Rovalpituzumab tesirine resistance: analysis of a corresponding small cell lung cancer and circulating tumor cell line pair. Anticancer Drugs. (2022) 33:300–7. 10.1097/CAD.0000000000001267 34924498

[37] Cortés J Kim SB Chung WP Im SA Park YH Hegg R. Trastuzumab Deruxtecan versus Trastuzumab Emtansine for Breast Cancer. N Engl J Med. (2022) 386:1143–54. 10.1056/NEJMoa2115022 35320644

[38] Desai A Abdayem P Adjei AA Planchard D. Antibody-drug conjugates: a promising novel therapeutic approach in lung cancer. Lung Cancer. (2022) 163:96–106. 10.1016/j.lungcan.2021.12.002 34942494

[39] Slaney CY Wang P Darcy PK Kershaw MH. CARs versus BiTEs: A Comparison between T Cell-redirection strategies for cancer treatment. Cancer Discov. (2018) 8:924–34. 10.1158/2159-8290.CD-18-0297 30012854

[40] Johnson ML Dy GK Mamdani H Dowlati A Schoenfeld AJ Pacheco JM. Interim results of an ongoing phase 1/2a study of HPN328, a tri-specific, half-life extended, DLL3-targeting, T-cell engager, in patients with small cell lung cancer and other neuroendocrine cancers. (2022) 40:8566. 10.1200/JCO.2022.40.16_suppl.8566

[41] Chen X Amar N Zhu Y Wang C Xia C Yang X. Combined DLL3-targeted bispecific antibody with PD-1 inhibition is efficient to suppress small cell lung cancer growth. J Immunother cancer. (2020) 8:e000785. 10.1136/jitc-2020-000785 32554616 PMC7304844

[42] Isobe Y Sato K Nishinaga Y Takahashi K Taki S Yasui H. Near infrared photoimmunotherapy targeting DLL3 for small cell lung cancer. EBioMedicine. (2020) 52:102632. 10.1016/j.ebiom.2020.102632 31981983 PMC6992936

[43] Tully KM Tendler S Carter LM Sharma SK Samuels ZV Mandleywala K. Radioimmunotherapy Targeting Delta-like Ligand 3 in Small Cell Lung Cancer Exhibits Antitumor Efficacy with Low Toxicity. Clin Cancer Res. (2022) 28:1391–401. 10.1158/1078-0432.CCR-21-1533 35046060 PMC8976830

[44] Sharma SK Pourat J Abdel-Atti D Carlin SD Piersigilli A Bankovich AJ. Noninvasive Interrogation of DLL3 Expression in Metastatic Small Cell Lung Cancer. Cancer Res. (2017) 77:3931–41. 10.1158/0008-5472.CAN-17-0299 28487384 PMC5534176

[45] Ataee M Mirhosseini S Mirnejad R Rezaie E Hosseini H Amani J. Design of two immunotoxins based rovalpituzumab antibody against DLL3 receptor; a promising potential opportunity. Res Pharm Sci. (2022) 17:428. 10.4103/1735-5362.350243 36034078 PMC9400466

